# Membranes of MnO Beading in Carbon Nanofibers as Flexible Anodes for High-Performance Lithium-Ion Batteries

**DOI:** 10.1038/srep14146

**Published:** 2015-09-16

**Authors:** Xin Zhao, Yuxuan Du, Lei Jin, Yang Yang, Shuilin Wu, Weihan Li, Yan Yu, Yanwu Zhu, Qinghua Zhang

**Affiliations:** 1 College of Material Science & Engineering, State Key Laboratory for Modification of Chemical Fibers and Polymer Materials, Donghua University, Shanghai 201620, China; 2 National Engineering Research Center for Nanotechnology, No.28 East Jiangchuan Road, Shanghai, 200241, P. R. China; 3 Key Laboratory of Materials for Energy Conversion, Chinese Academy of Sciences, Department of Materials Science and Engineering, University of Science and Technology of China, 96 Jin Zhai Rd, Hefei, Anhui Province, 230026, P. R. China; 4 iChEM (Collaborative Innovation Center of Chemistry for Energy Materials), Hefei 230026, China

## Abstract

Freestanding yet flexible membranes of MnO/carbon nanofibers are successfully fabricated through incorporating MnO_2_ nanowires into polymer solution by a facile electrospinning technique. During the stabilization and carbonization processes of the as-spun membranes, MnO_2_ nanowires are transformed to MnO nanoparticles coincided with a conversion of the polymer from an amorphous state to a graphitic structure of carbon nanofibers. The hybrids consist of isolated MnO nanoparticles beading in the porous carbon and demonstrate superior performance when being used as a binder-free anode for lithium-ion batteries. With an optimized amount of MnO (34.6 wt%), the anode exhibits a reversible capacity of as high as 987.3 mAh g^−1^ after 150 discharge/charge cycles at 0.1 A g^−1^, a good rate capability (406.1 mAh g^−1^ at 3  A g^−1^) and an excellent cycling performance (655 mAh g^−1^ over 280 cycles at 0.5 A g^−1^). Furthermore, the hybrid anode maintains a good electrochemical performance at bending state as a flexible electrode.

Flexible lithium-ion batteries (LIBs) hold great promise for the next generation energy sources of future electronic devices. They have found a wide variety of promising applications including smart clothes, electronic skins and wearable sensors and so on^1^,^2^. As core components of flexible LIBs, flexible electrodes are usually made from various functional organic and/or inorganic materials build on/in film-like carbon based materials from carbon nanotubes (CNTs)^3^,^4^,^5^, graphene^6^,^7^,^8^, carbon cloth/textiles^9^,^10^,^11^,^12^, *etc*. Besides, a preferable mass loading of active materials is important to maximum their utilization in hybrids. Due to the low cost and easy processing, carbon nanofibers (CNFs) membrane has advantages for being used as a free-standing and flexible substrate to construct metal or metal oxides/carbon composites^13^. The electrospinning process followed by stabilization and carbonization has been proved to be a facile and controllable way for fabricating CNFs membranes^14^. Some CNFs-based systems, such like Ge/CNFs^15^, Se/CNTs^16^, Ti/CNFs^17^, MoS_2_/CNFs^18^ have been fabricated and investigated for flexible electrode of LIBs or Sodium-ion batteries (SIBs). Therefore, it is highly desired if a proper form of metal compound is introduced in CNFs *in*-*situ* during the fabrication of CNFs membranes.

Recently, Mn-based nanomaterials have been intensively studied for energy storage^19^,^20^,^21^. MnO has attracted much attention because of its high theoretical capacity (756 mAh g^−1^), low conversion potential (average discharge and charge voltages of 0.5 V and 1.2 V *vs.* Li/Li^+^, respectively), environmentally benign features and low cost^22^,^23^,^24^. To tackle its shortcomings of capacity fading and poor rate capability, some MnO-carbon hybrids have been developed, such as MnO/mesoporous carbon^25^,^26^, MnO/graphene^23^,^27^,^28^,^29^,^30^, MnO/carbon^21^ nanowires^31^,^32^,^33^ and MnO/CNTs^34^,^35^,^36^, *etc*. However, in most cases the aforementioned products were in powder. Although Huang’s group fabricated MnO/CNFs composite by an electrospinning technique, a polymeric binder was still used for the fabrication of the electrodes^31^. Herein, we demonstrated the synthesis of MnO nanoparticles (NPs) beading in CNFs membranes by introducing MnO_2_ nanowires (NWs) into the polyacrylonitrile (PAN) solution through electrospinning followed by stabilization and carbonization processes. Structural transformation from MnO_2_ NWs to MnO NPs was accompanied by the simultaneous conversion of amorphous polymer to porous graphitic CNFs. The obtained membranes with MnO NPs uniformly embedded in the porous CNFs could be used directly as binder-free electrode for flexible LIBs. Effects of the loadings of MnO NPs in CNFs on the electrochemical performance of the composites were also investigated. With a preferable loading of MnO (34.6 wt%), the MnO/CNFs (MnC) membrane exhibits a high reversible capacity of 987.3 mAh g^−1^ after 150 discharge/charge cycles at 0.1 A g^−1^, a good rate capability (406.1 mAh g^−1^ at 3  A g^−1^) and an excellent cycling performance (655 mAh g^−1^ over 280 cycles at 0.5 A g^−1^). Besides, the hybrid membrane shows good electrochemical performance at bending state, demonstrating a great potential as a high performance anode material for flexible LIB applications.

## Results

**Figure 1:**
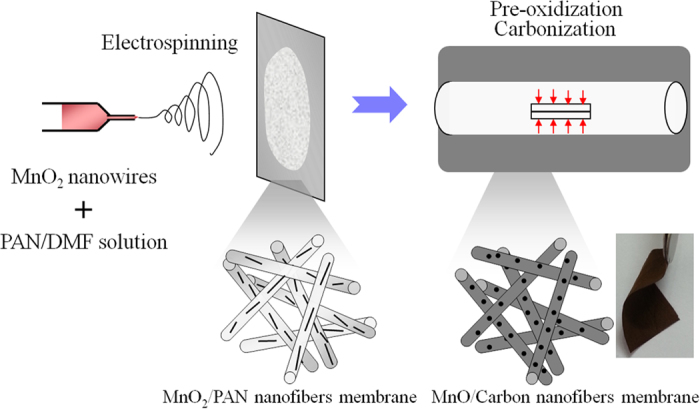
Schematic illustration for the preparation process of free-standing MnO/carbon nanofiber membranes.

**Figure 2:**
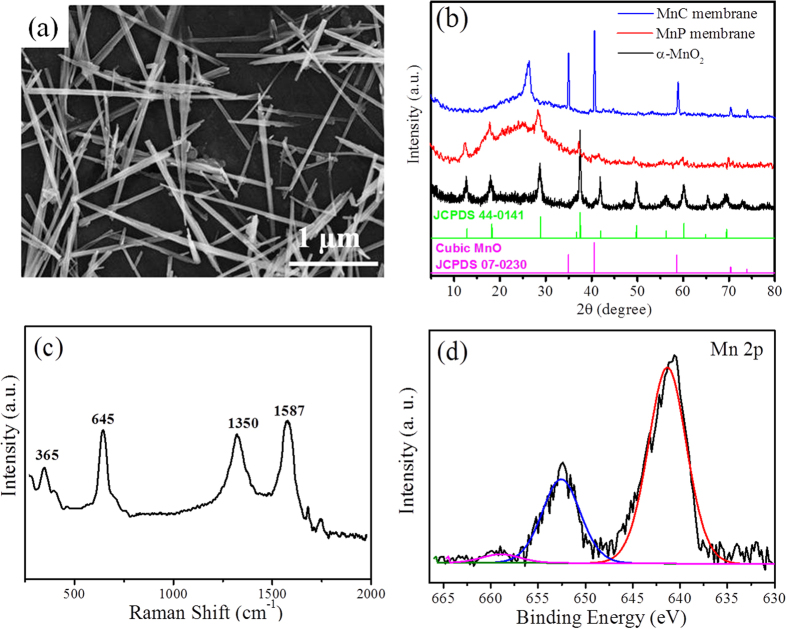
(**a**) SEM image of MnO_2_ NWs; (**b**) XRD patterns of MnO_2_ NWs and electrospun MnP, MnC membranes; (**c**) Raman spectra and (**d**) XPS Mn 2p spectra of MnC membrane.

Figure 1 illustrates the fabrication process of the flexible MnC membrane as reported previously^37^. Typically, a homogeneous blend solution with MnO_2_ nanostructures dispersed in PAN/ dimethylformamide (DMF) was electrospun into nanofibers, following with stabilization in air and a carbonization process in an inert gas. The fabricated MnO_2_/PAN nanofiber (MnP) membranes have a dark grey color due to the existence of MnO_2_, and the color is changed to black after carbonization. Different from our previous work^37^, in which a rod-like hierarchical core-corona nanostructure was prepared by a hydrothermal method, here we selected MnO_2_ NWs as Mn source, which are more likely uniaxially aligned along the CNFs. SEM image of pristine MnO_2_ NWs (Fig. 2(a)) displays monodispersed nanostructures with an average length of 1–2 μm and a diameter of 20–30 nm. The X-ray diffraction (XRD) pattern of MnO_2_ NWs (Fig. 2(b)) shows the typical diffraction peaks which can be indexed to α-MnO_2_ phase (JCPDS 44-0141). The peaks at 12.8^°^, 18.1^°^, 28.8^°^ and 37.5^°^ can be clearly seen from the as-spun MnP membrane, which are attributed to the diffractions from (110), (200), (310) and (211) faces of α-MnO_2_^38^,^39^, respectively, suggesting well-maintained crystal nanostructures of MnO_2_ NWs in MnP membrane. After carbonization, no α-MnO_2_ diffraction peaks but strong peaks at 35.0^°^, 40.6^°^, 58.6^°^ and 70.3^°^ are observed, assigned to the (111), (200), (220) and (311) reflections of cubic MnO (JCPDS 78-0424), respectively. Accompanying with the structural transformation from α-MnO_2_ to MnO crystals, the PAN has been carbonized to carbons at the high temperature in an inert atmosphere, as evidenced by the diffraction peak appearing at around 26.5^°^ in the XRD spectra of MnC membrane. Comparing with the XRD curve of graphite (Figure S1), which shows a very sharp peak at around 26.5^°^, the obtained carbon nanofibers here is believed to be composed of amorphous and graphitic carbon.

Raman spectroscopy was further used to confirm the existence of MnO and carbon (Fig. 2(c)). The signals observed at 365 and 645 cm^−1^ can be ascribed to Mn-O vibrations according to the literatures^23^. Two peaks at around 1350 and 1587 cm^−1^ are obtained, corresponding to D and G bonds of graphitic carbon, respectively^40^. The presence of D band suggests the existence of defects in the sample, which has been well characterized for more Li storage sites than graphitic carbon^41^. The X-ray photoelectron electroscopy (XPS) spectrum of MnC membrane (Figure S2 (a)) confirmed the presence of Mn, O and C elements and the C 1 s spectrum (Figure S2 (b)) could be decomposed into six components including C(sp^2^) (286.4 eV), C(sp^3^) (285.4 eV), C-O (286.1 eV), C = O (287.8 eV), O-C = O(298.5 eV) and π-π*(291.7 eV). The two signals at 641.4 eV and 562.6 eV from the Mn 2p XPS spectrum (Fig. 2(d)) could be attributed to Mn(II)2p_3/2_ and 2p_1/2_, respectively, characteristic of Mn in MnO^23^,^42^. Besides, a splitting satellite peak was found at 6.1 eV above the 2p_1/2_ principal peak, further indicating the existence of MnO^43^,^44^. Energy-dispersive spectroscopy (EDS) mapping images (Figure S3) also confirm the existence of Mn, O and C elements.

The MnO_2_ content in MnP membranes were tuned by adjusting the amount of MnO_2_ NWs in PAN/DMF solutions before electropinning; three samples are denoted here as MnP-1 (10 wt%), MnP-2 (20 wt%) and MnP-3 (30 wt%). The corresponding MnC membranes are designated as MnC-1, MnC-2 and MnC-3, respectively. The mass loading of MnO in the final products was estimated by thermogravimetric analysis (TGA, Figure S4). The mass loss from 150–400 °C is related to the combustion of carbon^24^ and the weight increase from 700–900 °C is due to the reaction: 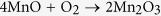
41,45. Considering the weight increase from MnO to Mn_2_O_3_, the calculated contents of MnO in MnC were 15.8 wt%, 34.6 wt% and 50.6 wt% for MnC-1, MnC-2 and MnC-3, respectively. The lower temperature of carbon combustion for samples with higher MnO contents can be explained by the catalytic function of the Mn species in the composites^46^.

**Figure 3:**
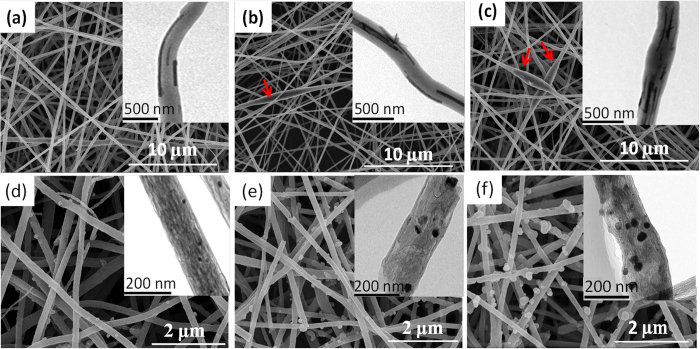
SEM images and TEM images (inset) for **(a)** MnP-1, **(b)** MnP-2, **(c)** MnP-3 and **(d)** MnC-1, **(e)** MnC-2, **(f)** MnC-3.

SEM and TEM images for MnP and MnC membranes are shown in Fig. 3. We can see that the as-spun nanofibers in MnP membranes (Fig. 2(a–c)) have uniform diameters of 200–300 nm and lengths of up to several micrometers. Before thermal treatment, MnO_2_ NWs are embedded in the co-axial nanofibers. Such a morphology has been observed from the samples with various loadings, except for some MnO_2_ NWs bundles being observed in MnP-2 and MnP-3 (insets of Fig. 3(b,c)). After carbonization, the morphologies and diameters of nanofibers in MnC membranes are well maintained. Interestingly, the MnO_2_ NWs are broken into MnO NPs with a typical diameter of 20–30 nm, leading to the formation of MnO NPs embedded in the CNFs (insets of Fig. 3(d,e)). Few additional particles were observed on the exterior surface of nanofibers for MnC-1 and MnC-2, but a large addition of MnO_2_ led to an accumulation of MnO NPs on the external structure for MnC-3 (Fig. 3(d)).

**Figure 4:**
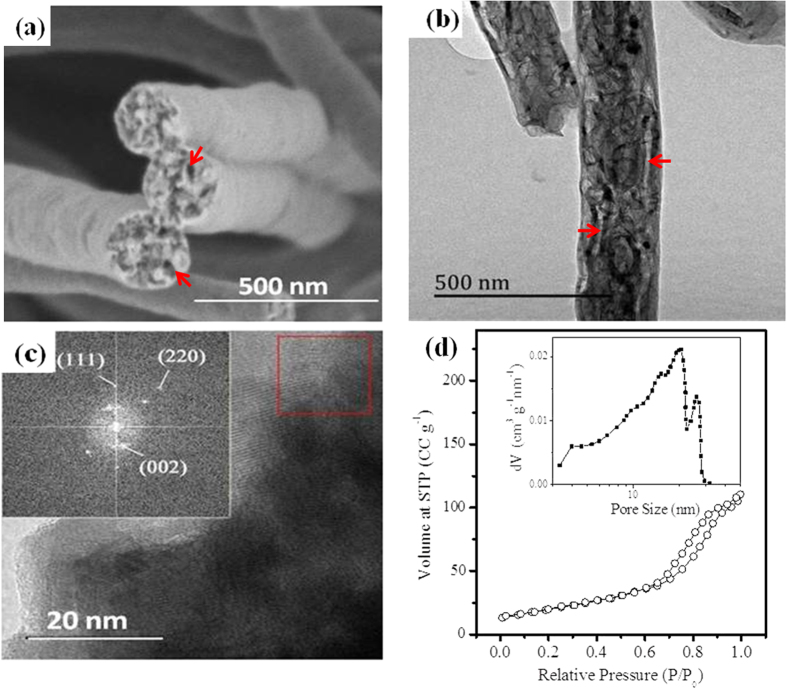
(**a**) SEM image of the cross section of MnC-2 membrane; (**c**) TEM image of MnC-2 membrane and its HRTEM micrograph with the corresponding FFT image of the red square area (inserted); (**d**) Nitrogen adsorption isotherms of MnC-2 membrane with its pore size distribution curve.

The cross-section view of nanofibers in MnC-2 membrane (Fig. 4(a)) displays a rough and porous structure with a pore diameter of about 20–30 nm. TEM image (Fig. 4(b)) further confirms the existence of the tubular pores inside nanofibers. The formation of such a porous structure could be attributed to the transformation of Mn-oxides especially accompanying the carbonization of PAN fibers, which is in favor of the access of electrolyte ions to active materials and of buffering the volumetric expansion during the Li^+^ insertion/extraction. N_2_ adsorption/desorption isotherm and the pore size distribution of MnC-2 (Fig. 4(d)) suggests that numerous mesopores are indeed present with diameters ranging between 20–50 nm. With such a mesoporous structure, the electrodes may provide a continuous network for fast electron/Li^+^ transportation and for tolerance of volumetric change, leading to a relatively high capacity and good cycling stability. The high resolution TEM image (Fig. 4(c)) shows clear lattice fringes for graphitic structure and Fast Fourier Transform (FFT) of the red square area demonstrates (002) of carbon and (220), (111) diffraction points of MnO, further indicating that the obtained product is dominant by MnO particles.

XRD was employed to investigate the structural evolution of Mn-oxides during the thermal treatment process. Several samples were obtained by stabilization at 280 °C in air for 2 h followed without/with carbonization at different temperatures (500–1000 °C) in N_2_ for 2 h (Figure S5). The sample obtained by stabilization at 280 °C alone shows the characteristic peak of MnO_2_. However, diffractions at 35.0^o^, 40.6^o^ and 58.7^o^ appear after carbonization at 500 °C, indicating the formation of MnO crystals. The MnO signals become stronger and sharper with increase of temperature, suggesting the improved crystallinity of MnO. The diffraction peaks around 26.5^o^ becomes notable after 900 ^o^C, indicating the formation of graphitic carbon. In a control experiment, MnO_2_ NWs was treated under the same condition without PAN fibers. Large MnO particles with diameters of 200–300 nm were obtained (Figure S6), dramatically different from that of MnC. The structural transformation of MnO_2_ NWs is significantly different when PAN fibers are present. It is known that stabilization and carbonization are two vital steps taking place during the thermal treatment of PAN-based CNFs: a ladder-like structure could be formed at the early stabilization stage with dehydrogenation and cyclization reactions, and then an aromatic growth and polymerization occur to generate a turbostratic structure^14^. Here, in our work, the resultant MnO NPs embedded in porous CNFs might be related to the strong synergistic effects between the morphological evolution of Mn-oxides and the structural transformation of PAN from an amorphous to graphitic carbons.

**Figure 5:**
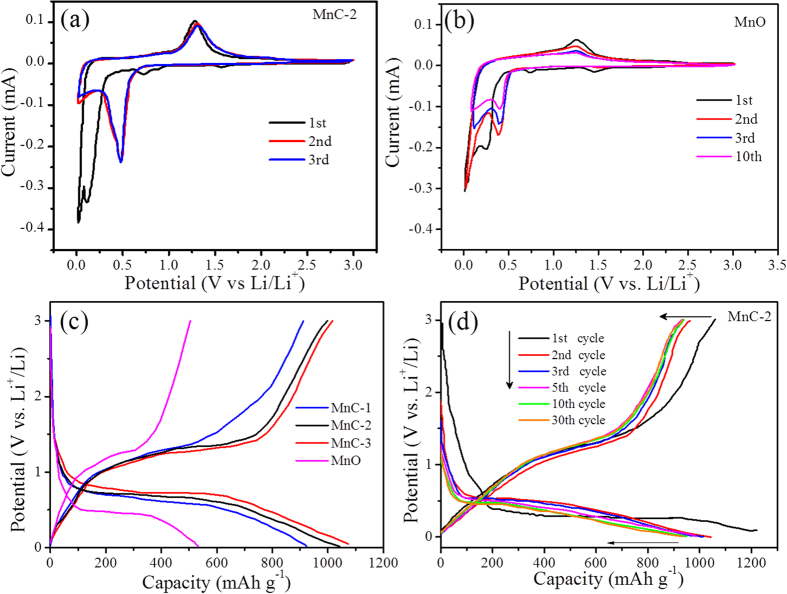
Electrochemical performance of the as prepared electrodes: **(a)** CV curves of MnC-2 and **(b)** MnO electrode at a scan rate of 0.2 mV s^−1^ for the initial several cycles; **(c)** discharge/charge profiles at a current density of 100 mA g^−1^ for different samples and **(d)** for MnC-2 at different cycles.

Free-standing MnC membranes have been directly used as anodes in coin-type Li half-cells and their electrochemical performance were investigated. The initial cyclic voltammograms (CV) curve of MnC electrode (Fig. 5(a)) shows an irreversible reduction peak at around 0.72 V in the first cycle, corresponding to the irreversible reduction of electrolyte and the formation of a solid electrolyte interphase (SEI) layer^47^,^48^. Another obvious cathodic peak close to 0.11 V is attributed to the initial reduction of Mn^2+^ to metallic Mn^0^ (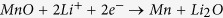), which significantly shifts to 0.48 V in the following cycles. The shift is probably due to the enhanced kinetics and enhanced utilization efficiency of MnO NPs in the hybrid electrode, arising from the microstructure alternation after the first lithiation^30^,^49^. During the anodic scan, the oxidation peak at about 1.29 V can be ascribed to oxidation of Mn^0^ to Mn^2+^ (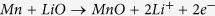), which slightly shifts to 1.31 V. The second and onward CV curves indicate a good reversibility and structural stability of MnC during the electrochemical process. On the contrary, those of bare MnO NPs (Fig. 5(b)) display a reducing tendency in capacity for the initial several cycles.

The discharge/charge curves of the MnC and MnO electrodes (Fig. 5(c)) demonstrate a higher specific capacity of MnC than that of MnO and a larger value of MnC-3 with a higher content of MnO. As summarized in Table 1, the initial discharge capacities of MnC-1, MnC-2 and MnC-3 in the first cycle are 1114.4, 1220.0 and 1363.6 mAh g^−1^ respectively, indicating a high accessibility for Li^+^ insertion/extraction in the MC electrodes. In addition, the initial columbic efficiency (CE) of MnC-1, MnC-2 and MnC-3 is 80.7%, 86.1% and 72.3% respectively, higher than that of MnO (69.1%) and the reported MnO/carbon and MnO/graphene composites^27^,^29^,^41^. The results show that the stable structure of CNFs in our MnC membranes could effectively reduce the pulverization of MnO and etching by electrolyte, thus limiting the production of thicker SEI layer during the initial discharge/charge process^50^. A lower CE of MnC-3 than that of MnC-1 or MnC-2 could be attributed to the unfavorable formation of SEI layers caused by the aggregation of MnO NPs. The discharge/charge curves (Fig. 5(d)) for MnC in the different cycles at 0.1 A g^−1^ demonstrate that the hybrids gradually reach a stable status in the second and subsequent cycles.

**Figure 6:**
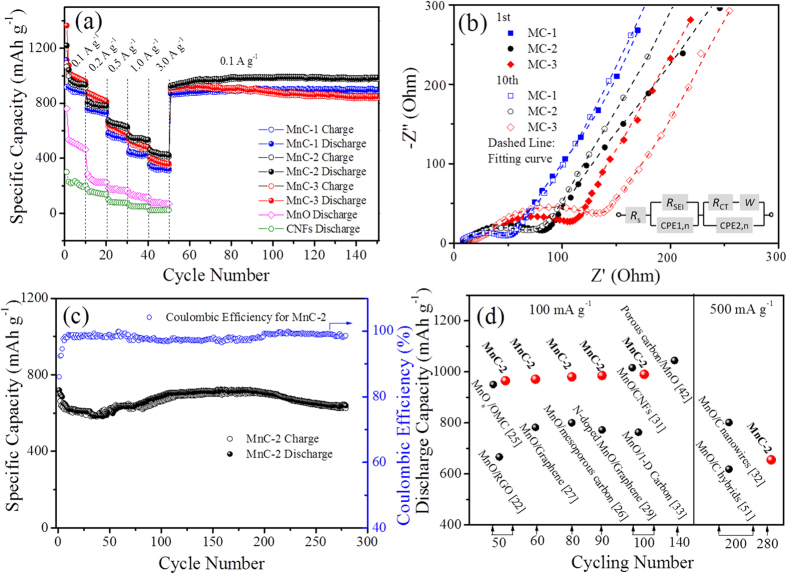
(**a**) rate capacities at different current densities and capacity at different cycles for MnC and MnO; (**b**) Nyquist curves of MnC samples at 1^st^ and 10^th^ cycle and their respective fittings with an appropriate electric equivalent circuit; (**c**) cycling performance of MnC-2 with columbic efficiency; (**d**) a comparison of the discharge capacities of MnC-2 with reported MnO-based materials at various cycles.

The rate performance was investigated by increasing the current density stepwise from 0.1 A g^−1^ to 3 A g^−1^ and subsequently coming back to 0.1 A g^−1^ and then the cycling performance were evaluated by the following 100 discharge/charge cycles at 0.1 A g^−1^ (Fig. 6(a)). It is obvious that MnC electrodes displays much higher capacities than that of MnO (Figure S7) and MnC-2 shows a better rate performance although it processes lower discharge capacities in the first two stages (0.1 and 0.2 A g^−1^) than that of MnC-3. The average reversible capabilities of MnC-2 are 918.7, 785.4, 610.6 and 532.2 mAh g^−1^ at 0.1 A g^−1^, 0.2 A g^−1^, 0.5 A g^−1^ and 1 A g^−1^, respectively. Even at a high rate of 3 A g^−1^, the discharge capacity of 406.1 mAh g^−1^ remains about 44.1% of the initial value at 0.1 A g^−1^. While MnC-3 and MnC-1 sample only retain 39.5% and 38%, respectively. When the current density is switched back to 0.1 A g^−1^ after 50 cycles at various current densities, a reversible capacity of 927.1 mAh g^−1^ is recovered for MnC-2, higher than its initial value at the same rate. However, MnC-3 only delivers 911.6 mAh g^−1^ in the same case. Furthermore, in the following cycling tests at 0.1 A g^−1^, a reversible capacity of as high as 987.3 mAh g^−1^ are obtained for MnC-2 but the value of MnC-3 falls to 898.1 mAh g^−1^. As shown in Fig. 3, the aggregation of MnO NPs protruding out of the external structure for MnC-3 might reduce the conductivity of the electrode and weaken the cushion effects of carbon materials, thus leading to a declined capacity. Moreover, MnC-1 and MnC-2 display reduced charge transfer resistance (*R*_ct_) after 10 cycles (smaller than that of MnO, Figure S8), but a large increase of SEI layer (*R*_SEI_) and *R*_ct_ of MnC-3 is obtained from the Nyquist plots after cycling (Fig. 6(b) and Table S1), suggesting MnC-3 possessing continuous formation of SEI layer and a slower charge transfer rate.

The cycling curve of MnC-2 at 0.5 A g^−1^ and the corresponding CE value (Fig. 6(c)) further demonstrate that it possesses an excellent cycling performance with a columbic efficiency of ~99%. It should be noted that the capacity of MnC-2 firstly drops and then increases upon cycling, which is probably due to the activation process during the discharge/charge process for MnO/C hybrids^20^,^30^,^39^,^47^. After 280 cycles, the reversible capacity retains a high value of 655 mAh g^−1^, higher than the reported value for porous MnO/C core/shell hybrids, e.g., 618.3 mAh g^−1^ after 200 cycles at 0.5 A g^−1^ 51. A comparison of the capacities of MnC-2 with those of reported MnO-based materials after various cycles (Fig. 6(d)) clearly shows that out MnC hybrids display a better cycling performance. Furthermore, SEM and TEM images after cycling turn out to be a direct proof of the structural stability and integrity of the materials (Figure S9). No pulverization or obvious size variation is observed, indicating that such hybrids can indeed relieve the strain and stress caused by volume variation and prevent the aggregation or detachment of inner MnO NPs over cycling process.

## Discussion

**Figure 7:**
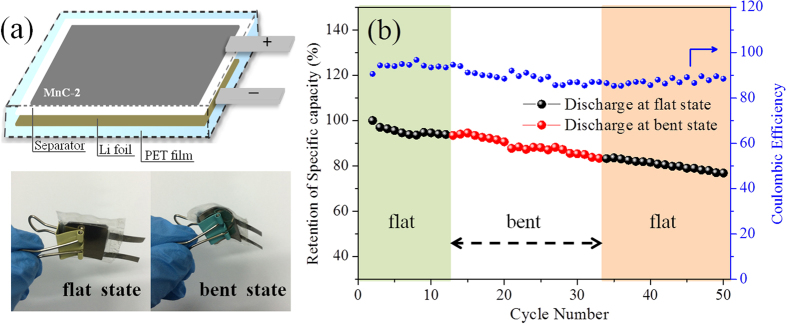
(**a**) Scheme of the assembled flexible cell encapsulated by PET film and photograph of the battery tested at flat and bent states; (**b**) cyclic performance of the battery under flat and bent states.

As discussed, with appropriate contents of MnO NPs, *i.e.*, 34.6 wt% in our work, a favorable porous structure with the active materials being well confined in CNFs are obtained, leading to an improvement of Li reaction kinetics and giving rise to a comprehensively improved electrochemical properties including high specific capacities, rate performance and cycling stability. Several unique characteristics of the MnC membrane could contributed to the high performance anode material in LIBs: (i) the conductive networks arising from CNFs increases the conductivity of the electrode and provide fast transfer paths for electrons and Li ions; (ii) the porous structure of MnC promotes the diffusion of the liquid electrolyte into the bulk of anode materials, thus improving the efficient utilization of the active materials and giving rise to high capacities; (iii) the architecture with MnO NPs well beading in CNFs is in favor of relieving the strain induced by the volume change during the charge/discharge cycles, leading to an enhanced cycle stability. Furthermore, to investigate the potential of as-prepared samples for use as flexible electrodes, a flexible cell was assembled and tested to obtain the effect of bending on the electrochemical performance (Fig. 7). It is obvious that the capacity of the battery being bent have a negligible decrease compared with that of the original flat battery. Moreover, the flexible battery showed a good cyclic stability both under flat and bent states. With respect to the original capacity, it showed a capacity retention of ~93.8% after the first 12 cycles under a flat state, and ~84% after another 20 cycles under a bent state. Such a facile and practical fabrication process of MnC membranes might be an efficient route to design and fabricate high performance flexible electrode materials for LIBs.

In summary, we have successfully fabricated MnC membranes through incorporating MnO_2_ NWs into the electrospun solution by a facile and practical electrospinning technique followed by stabilization and carbonization processes. During the thermal treatment, structural changes from MnO_2_ NWs to MnO NPs occurred accompanying with the conversion from an amorphous state of polymer to a graphitic structure of CNFs. The resultant MnC architecture with MnO NPs being well embedded in the porous carbon structures not only facilitates the transport of both electrolyte ions and electrons to the electrode surface, but also enhances the utilization of the active materials. With suitable amounts of MnO NPs, the resultant flexible MnC membrane demonstrates excellent electrochemical properties with high specific capacitie of 987.3 mAh g^−1^ after 150 discharge/charge cycles at 0.1 A g^−1^, good rate performance (406.1 mAh g^−1^ at 3 A g^−1^) and cycling stability (655 mAh g^−1^ over 280 cycles at 0.5 A g^−1^). We believe that such low cost, high-performance hybrids fabricated by earth-abundant and environmentally friendly materials and scalable electrospinning techniques can offer a great promise in grid-scale electrode materials for flexible LIBs.

## Experimental Section

### Materials

Polyacrylonitrile (PAN, M_w_ = 80000) was made in laboratory^52^. Potassium permanganate (KMnO_4_, AR), sulfuric acid (H_2_SO_4_, AR) and N,N-dimethylformamide (DMF, AR) were purchased from Shanghai Lingfeng Chemical Reagent Co., Ltd. Manganese sulfate monohydrate (MnSO_4_·H_2_O, AR) was purchased from Sinopharm Chemical Reagent Co., Ltd. All these reagents were used without further purification.

### Preparation of MnO_2_ nanowires (MnO_2_ NWs)

MnO_2_ NWs were synthesized by a hydrothermal method. Briefly, aqueous solutions of MnSO_4_·H_2_O (1 mmol) and KMnO_4_, (1.5 mmol) (Mn(II)/Mn(VII) = 3:2) were mixed with vigorously stirring. The PH value of the mixture was adjusted ~2 with 5 M H_2_SO_4_ aqueous solution. Then the solution was transferred to an autoclave and reacted in oven at 140 °C for 12 h. After cooling down, the product was collected by filtration and washed repeated with distilled water and absolute ethanol. Then the MnO_2_ NWs powder was obtained.

### Preparation of MnO_2_ NWs/PAN nanofiber (MnP) membranes

The obtained MnO_2_ NWs powder was washed three times with DMF, centrifuged and then added into a certain-mass 8 wt% PAN/DMF solution as an electrospinning solution. Intensive stirring was conducted for 12 h in order to get a homogeneously distributed solution. Then the blended solution was electrospun into MnP membranes, with a controlled syringe pump of 25 μL min^−1^ and an applied voltage of 20 kV with a distance of 20 cm between the electrospinning jet and the collector. The adding amounts of MnO_2_ NWs based on PAN were 10 wt%, 20 wt% and 30 wt% for MnP-1, MnP-2 and MnP-3, respectively.

### Preparation of MnO/carbon nanofiber (MnC) membranes

The as-prepared MnP membranes were firstly performed at 280 °C in air for 2 h for a stabilization process. Next, the temperature increased to 1000 °C with a heating rate of 3 °C/min and stayed for 1 h in N_2_ atmosphere. Then the MnC membranes were obtained after the furnace cooling down to the room temperature. During both the stabilization and carbonization processes, the membranes were sandwiched between two aluminum oxide (Al_2_O_3_) plates to give certain tensions against the planer dimensional shrinkage. For the controlled experiment, MnO_2_ NWs powder was treated with the similar procedure in a Al_2_O_3_ container.

### General characterization

The structure of the obtained samples was characterized by X-Ray diffraction (XRD) measurement was conducted on a Rigaku D-max-2500 diffractometer with nickel-filtered Cu- Kα radiation with λ = 1.5406 Å. The morphology and microstructure of the composites were characterized by field emission scanning electron microscopy (FESEM, Hitachi S-4800) in conjunction with energy dispersive X-ray spectroscopy (EDS) and transmission electron microscopy (TEM, JEOL JEM-2100) at an accelerating voltage of 100 kV. Thermogravimetric analysis (TGA, Perkin-Elmer TGA 4000) was measured with a heating rate of 5 °C/min under 20 mL/min of flowing air. X-ray photoelectron spectroscopy (XPS) experiment was carried out on a RBD upgraded PHI-5000C ESCA system (Perkin Elmer) with Mg Kα radiation (hν = 1253.6 eV). The measurement of the nitrogen adsorption isotherms was done with a Quantachrome Nova 2000 at 77. 4 K. Raman spectra were recorded using a LabRam -1B Ramen spectroscope with He-Ne laser excitation at 632.8 nm and scanning for 50 s.

### Electrochemical characterization

The obtained MnC membranes were punched into 1 cm diameter electrodes and could be used directly. For MnO electrode, homogeneous slurry composed of MnO powder, poly(vinyl difluoride) (PVDF) and acetylene black in N-methyl-2-pyrrolidinon (NMP) with a weight ratio of 60:10:30 was prepared under magnetic stirring for 12 hours and then was coated onto clean copper foil (∼10 μm) current collector. All the electrodes were firstly dried in a vacuum oven at 60 °C overnight and then assembled into LR 2032 type coin cells in an argon-filled glove box. The electrolyte was 1 M solution of LiPF_6_ in ethylene carbonate (EC) and dimethyl carbonate (1:1, v/v). Microporous polypropylene sheet (Celgard, 2400) was used as the separator. The galvanostatic discharge/charge cycles of the cells were performed over the potential range between 0.01 and 3.0 V on Land instrument (CT2001A). Cyclic voltammetry (CV) and the electrochemical impedance spectroscopy (EIS) were performed on Autolab PGSTAT 302N electrochemical workstation with a scan rate of 0.2 mV s^−1^ from 0.01 V to 3 V and by applying a perturbation voltage of 10 mV in a frequency range of 100 kHz to 10 mHz at the open circuit potential, respectively.

## Additional Information

**How to cite this article**: Zhao, X. *et al.* Membranes of MnO Beading in Carbon Nanofibers as Flexible Anodes for High-Performance Lithium-Ion Batteries. *Sci. Rep.* **5**, 14146; doi: 10.1038/srep14146 (2015).

## Supplementary Material

Supplementary Information

## Figures and Tables

**Table 1:** The electrochemical properties of various anode materials.

Samples	Discharge capacity (mAh g^−1^)	1^st^ cycle	0.1 mA g^−1^	10^th^ cycle	51^th^ cycle	150^th^ cycle
		Charge capacity (mAh g^−1^)	Columbic efficiency	Discharge capacity (mAh g^−1^)		
MnC-1	1114.4	899.3	80.7%	873.1	897.0	910.6
MnC-2	1220.0	1050.3	86.1%	918.7	927.1	987.3
MnC-3	1363.6	986.6	72.3%	956.6	911.6	898.1
