# Supplementary material for: Membranes of MnO Beading in Carbon Nanofibers as Flexible Anodes for High-Performance Lithium-Ion Batteries
Source: Sci Rep. 2015 Sep 16;5:14146. doi: 10.1038/srep14146 (PMC4570985; doi:10.1038/srep14146)
Supplement: Supplementary Information [file srep14146-s1.doc]

**Supplementary Information**

**Membranes of MnO Beading in Carbon Nanofibers as Flexible Anodes for High-Performance Lithium-Ion Batteries**

Xin Zhao^1^, Yuxuan Du^1^, Lei Jin^1^, Yang Yang^2^, Shuilin Wu^3^, Weihan Li^3^, Yan Yu^3^, Yanwu Zhu*^3,4^ and Qinghua Zhang*^1^

*^1^College of Material Science & Engineering, State Key Laboratory for Modification of Chemical Fibers and Polymer Materials, Donghua University, Shanghai 201620, China*

*^2^National Engineering Research Center for Nanotechnology, No.28 East Jiangchuan Road, Shanghai, 200241, P.R. China*

*^3^Department of Materials Science and Engineering & CAS Key Laboratory of Materials for Energy Conversion, University of Science and Technology of China, and Collaborative Innovation Center of Chemistry for Energy Materials (2011-iChEM), Hefei 230026, China.*

*^4^iChEM (Collaborative Innovation Center of Chemistry for Energy Materials), Hefei 230026, China.*

*Corresponding author. Email: [zhuyanwu@ustc.edu.cn](mailto:zhuyanwu@ustc.edu.cn), [qhzhang@dhu.edu.cn](mailto:qhzhang@dhu.edu.cn)

**Figure S1.** XRD curves of graphite and MnC membrane.

**Figure S2.** XPS spectra and C 1s spectrum of MnC-2 membrane.

**Figure S3**. SEM image (a) combined with EDS profiles with the atomic calculation (b) and EDS mapping with the relative intensities of C (green), O (purple) and Mn (blue) elements (c) for MnC-2 electrodes and EDS profiles for the three samples.

**Figure S4**. TGA curves of various MnC-membranes and carbon nanofiber membranes.


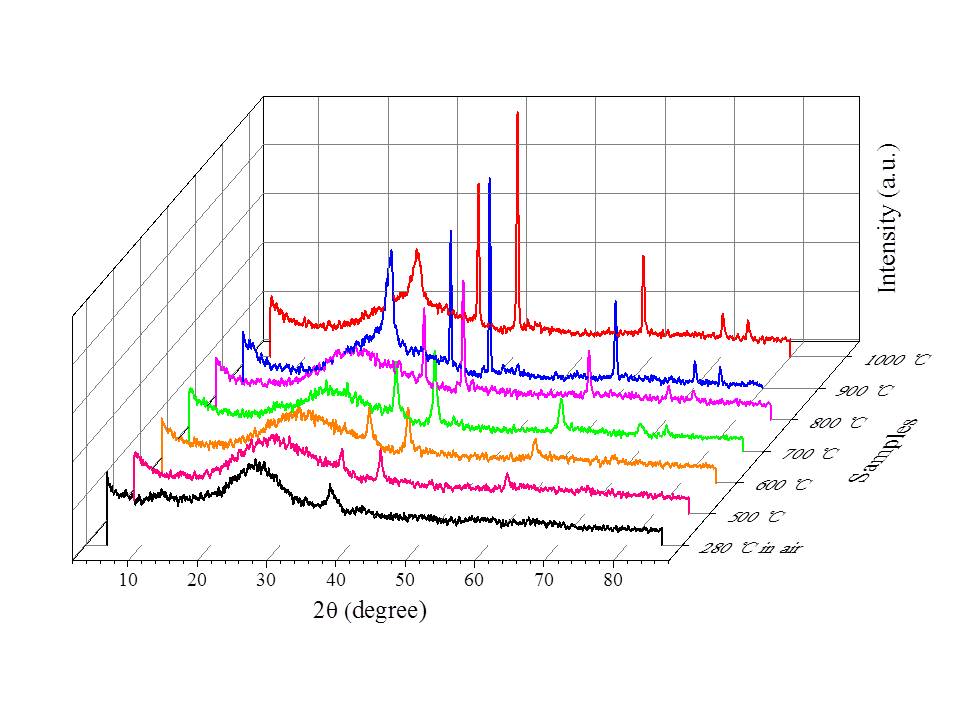


**Figure S5.** XRD spectra of several samples prepared by stabilization at 280 ℃ in air without and with carbonization at different temperature (500-1000 ℃)

**Figure S6**. SEM image and XRD spectra of as prepared MnO powder.

**Figure S7**. (a) CV curve at 0.2 mV s^-1^, (b) rate performance, (c) cycling stability at 0.5 mV s^-1^ and (d) Nyquist polt of bare CNFs electrode

**Figure S8**. Nyquist plots of MnC-2 and MnO electrodes

**Figure S9**. The post-mortem SEM (a) and TEM images (b) of MnC-2 electrode after cycling test.

**Table S1**. The fitting results of various anodes based on electric equivalent circuit at different cycles

| samples | 1^st^ cycle | | | 10^th^ cycle | | |
| --- | --- | --- | --- | --- | --- | --- |
|  | *R*_s_ (Ohm) | *R*_SEI_(Ohm) | *R*_ct_(Ohm) | *R*_s_(Ohm) | *R*_SEI_(Ohm) | *R*_ct_(Ohm) |
| MnC-1 | 3.1 | 18.3 | 36.7 | 4.6 | 21.4 | 35.2 |
| MnC-2 | 4.9 | 27.6 | 50.2 | 4.1 | 33.6 | 46.5 |
| MnC-3 | 7.6 | 41.3 | 77.4 | 11 | 75.1 | 124.4 |
